# Modeling Schistosomiasis and HIV/AIDS Codynamics

**DOI:** 10.1155/2011/846174

**Published:** 2011-02-13

**Authors:** S. Mushayabasa, C. P. Bhunu

**Affiliations:** ^1^Modelling Biomedical Systems Research Group, Department of Applied Mathematics, National University of Science and Technology, P.O. Box 939 Ascot, Bulawayo, Zimbabwe; ^2^Department of Veterinary Medicine, University of Cambridge, Cambridge CB3 OES, UK

## Abstract

We formulate a mathematical model for the cointeraction of schistosomiasis and HIV/AIDS in order to assess their synergistic relationship in the presence of therapeutic measures. Comprehensive mathematical techniques are used to analyze the model steady states. The disease-free equilibrium is shown to be locally asymptotically stable when the associated disease threshold parameter known as the basic reproduction number for the model is less than unity. Centre manifold theory is used to show that the schistosomiasis-only and HIV/AIDS-only endemic equilibria are locally asymptotically stable when the associated reproduction numbers are greater than unity. The impact of schistosomiasis and its treatment on the dynamics of HIV/AIDS is also investigated. To illustrate the analytical results, numerical simulations using a set of reasonable parameter values are provided, and the results suggest that schistosomiasis treatment will always have a positive impact on the control of HIV/AIDS.

## 1. Introduction

Schistosomiasis, also known as bilharzia after Theodor Bilharz who first identified the parasite in Egypt in 1851, is a disease caused by blood flukes [1]. It affects millions of people worldwide, especially in South America, the Middle East, and Southeast Asia where it remains a public health problem and poses a threat to 600 million people in more than 76 countries [1]. The disease is often associated with water resource development projects, such as dams and irrigation schemes, where the snail intermediate hosts of the parasite breed [2]. Human schistosomiasis (which has a relatively low mortality rate, but a high morbidity rate) is a family of diseases primarily caused by three species of the genus* Schistosoma* or flat worms. The adult worms inhabit the blood vessels lining either the intestine or bladder, depending on the species of the worm [3]. The highest number of human schistosomiasis infections is caused by *S. haematobium*, which has a predilection for the blood vessels around the bladder and causes urinary disease [4]. Schistosomiasis is the second most prevalent neglected tropical diseases after hookworm (192 million cases), accounting for 93% of the world's number of cases and possibly associated with increased horizontal transmission of HIV/AIDS [5].

On the other hand, the number of people living with HIV worldwide continued to grow in 2008, reaching an estimated 33.4 million, which is more than 20% higher than the number in 2000, and the prevalence was roughly threefold higher than in 1990 [6]. The HIV virus, by holding the immune system hostage, has opened many gates for pathological interactions with other diseases [7]. Schistosomiasis and HIV infections have major effects on the host immune response, and coinfection (of the two diseases which may increase the complexity of treatment for people living with HIV and may contribute to poorer medical outcomes) is widespread [8]. While schistosomiasis infections are caused by diverse species from three phyla, HIV is essentially a single entity. There is some evidence that schistosomiasis infection provides some benefit in some instances like the atopic disease [9, 10], and the inflammatory pathology of autoimmune disease [11–13]. For bacterial and viral infections, impaired control of replication and elimination may lead to a detrimental outcome [14–17]. That HIV infection is detrimental to the immune response to many pathogens is quite clear and poor regulation of immune system in advanced HIV infection is illustrated by an increased incidence of hypersensitive drug reactions [18, 19]. Studies that examine the codynamics of HIV and schistosomiasis infections have shown a significant association between HIV and the presence of *S. haematobium* eggs in the genital samples, supporting the argument that schistosomiasis infection enhances HIV susceptibility when genital lesions are present [20]. Host-parasite interactions such as schistosomiasis, where inflammatory responses have persisted through evolution, perhaps due to selective advantage for parasite egg excretion, may be more detrimental with regard to HIV infection [21].

Although the negative impact of the synergetic interactions between HIV and schistosomiasis has shown to be a public health burden, only few statistical or mathematical models have been used to explore the consequences of their joint dynamics at the population level. There are plenty of single disease dynamic models. A significant number focus on HIV/AIDS [22–26] or on the transmission dynamics of schistosomiasis [27–37]. Schistosomiasis model (24) considered in this study differs from those found in the literature in that we consider* Schistosoma mansoni* a human blood fluke which causes schistosomiasis and is the most widespread and the fresh water snail* Biomphalaria glabrata* serves as the main intermediate host, while the HIV/AIDS model (7) is an extension of the model by Murray [38] by including HIV therapy while neglecting the issue of seropositivity considered in [38]. Mathematical modeling assessing the impact of schistosomiasis on the transmission dynamics of HIV/AIDS is rare [39].

Quantifying by how much treatment of schistosomiasis affects HIV/AIDS dynamics will require an extensive sensitivity analysis with parameter values estimated from real and recent coinfection data. Nevertheless, this theoretical study provides a framework for the potential benefit of schistosomiasis treatment on the dynamics of HIV and highlights the fact that global public health challenges require comprehensive and multipronged approaches to dealing with coinfections [7], and current intervention efforts that focus on a single infection at a time may be losing substantial rewards of dealing synergistically and concurrently with multiple infectious diseases in one host. To the best of our knowledge, except for the study in [39] where the co-interaction of schistosomiasis and HIV without any form of treatment is investigated, this work is possibly the first to give a theoretical mathematical account of the impact of schistosomiasis on HIV dynamics in the presence of both schistosomiasis treatment and antiretroviral therapy at the population level.

The rest of the paper is structured as follows. In the next section, we present the schistosomiasis and HIV/AIDS coinfection model. In Section 3 we determine sufficient conditions for local stability of the disease-free and endemic equilibria and analyze the reproduction number for the two diseases separately while Section 4 provides a comprehensive analysis of the full model.  Section 5 provides numerical results while Section 6 concludes the paper.

## 2. Model Description

The proposed model is an extension of an earlier study [39], which did not account for any intervention strategy. The schistosomiasis and HIV models will be coupled via the force of infection, and in the absence of any of the diseases (hence no coinfection), the two basic disease submodels can be decoupled from the general model (see Sections 3.1.4 and 3.3.1). The population of interest is divided into several compartments dictated by the epidemiological stages (disease status), namely, susceptibles *S*_*H*_(*t*), who are not yet infected by either HIV or schistosomiasis, schistosomiasis-infected individuals *I*_*B*_(*t*), HIV-infected individuals not yet displaying symptoms of AIDS *I*_*H*_(*t*), individuals infected with HIV showing symptoms of AIDS *A*_*H*_(*t*), individuals dually infected with schistosomiasis and HIV displaying symptoms of schistosomiasis only *I*_*H*_*B*__(*t*), individuals dually infected with schistosomiasis and HIV displaying symptoms of schistosomiasis and AIDS *A*_*H*_*B*__(*t*), treated individuals infected with HIV only, showing symptoms of AIDS *A*_HT_*A*__(*t*), and treated individuals dually infected with schistosomiasis and HIV displaying symptoms of schistosomiasis and AIDS *A*_*H*_*T*_*B*___(*t*). Other important populations to consider in this model are the susceptible snails *S*_*s*_(*t*), infected snails *I*_*s*_(*t*), miracidia population *M*(*t*), and the cercariae population *P*(*t*). Individuals move from one class to the next as the disease progresses and/or through dual infection. We further make the following assumptions for the model.

- There is no vertical transmission of both infections in humans.
- Infected snails do not reproduce due to castration by miracidia.
- Seasonal and weather variations do not affect snail populations and contact patterns.
- Susceptible humans become infected with schistosomiasis only through contact with free-living pathogen in infested waters.

At any time, new recruits enter the human and snail populations through birth/migration at constant rates Λ_*H*_ and Λ_*S*_, respectively. There is a constant natural death rate *μ*_*H*_ in each human subclass. The force of infection associated with HIV infection, denoted by *λ*_*H*_, is given by

(1) $$\begin{array}{c} \begin{array}{c} \lambda_{H}\left(t\right)=\frac{\beta_{H}c\left[I_{H}+I_{H_{B}}+\eta \left(A_{H}+A_{H_{B}}\right)+\kappa \left(A_{H_{T_{A}}}+A_{H_{T_{B}}}\right)\right]}{N_{H}}, \end{array} \end{array}$$

with *β*_*H*_ being the probability of HIV transmission per sexual contact, *c* is the effective contact rate for HIV infection to occur, and *η* > 1 models the fact that individuals in the AIDS stage and not on antiretroviral therapy are more infectious since the viral load is correlated with infectiousness [42]. It is assumed that individuals on antiretroviral therapy transmit infection at the smallest rate *κ* (with 0 < *κ* < 1) because of the fact that these individuals have very small viral load. It has been estimated by an analysis of longitudinal cohort data that antiretroviral therapy reduces per-partnership infectivity by as much as 60% (so that *κ* = 0.4) [41]. Thus, the total human population *N*_*H*_(*t*) is given by

(2) $$\begin{array}{cc} N_{H}\left(t\right) & =S_{H}\left(t\right)+I_{H}\left(t\right)+A_{H}\left(t\right)+A_{H_{T_{A}}}\left(t\right)\\ & \;+I_{\text{B}}\left(t\right)+I_{H_{B}}\left(t\right)+A_{H_{B}}\left(t\right)+A_{H_{T_{B}}}\left(t\right). \end{array}$$

Susceptible individuals acquire schistosomiasis following infection at a rate *λ*_*P*_, where

(3) $$\lambda_{P}=\frac{\beta_{P}P\left(t\right)}{P_{0}+\epsilon P\left(t\right)},$$

with *β*_*P*_ being the maximum rate of exposure, *ϵ* is the limitation of the growth velocity of cercariae with the increase of cases, and *P*_0_ is the half saturation constant. In the absence of the parasite, the functional response of individuals susceptible to the pathogen (schistosomiasis) is given by (*λ*_*P*_/*β*_*P*_)[*S*_*H*_(*t*) + *I*_*H*_(*t*) + *A*_*H*_(*t*) + *A*_*H*_*T*_*A*___(*t*)], a modified Holling's type-II functional response (also known as the Michaelis-Menten function when *ϵ* = 1), the response refers to the change in the density of susceptibles per unit time per pathogen as the schistosomiasis susceptible population density changes. From the functional response, we note that at low parasite density, contacts are directly proportional to host density, but a maximum rate of contact is reached at very high densities (saturation incidence). Individuals infected with schistosomiasis have an additional disease-induced death rate *d*_*B*_. Similarly, susceptible and infected snails have a natural death rate *μ*_*S*_, and the infected snails have an additional disease-induced death rate *d*_*S*_. The total snail population is given by *N*_*S*_(*t*) = *S*_*S*_(*t*) + *I*_*S*_(*t*).

**Figure 1:**
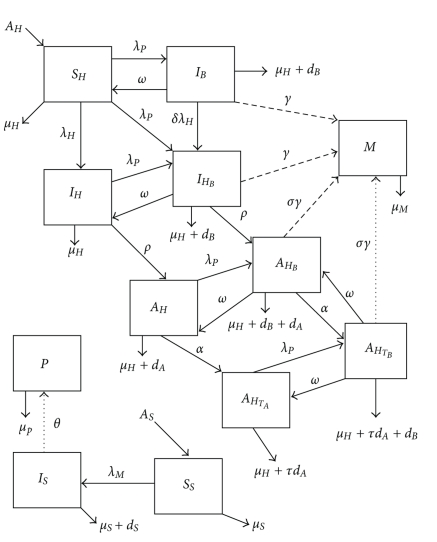
Model flow diagram.

 Considering a schistosomiasis-infected individual, a number (portion) *N*_*E*_ of eggs leave the body through excretion (faeces and urine) and find their way into the fresh water supply where they hatch into free swimming ciliated miracidium at a rate *γ* for individuals without AIDS. Given the weakened immune system of AIDS individuals, they tend to excrete more often, thus releasing more eggs which will hatch into miracidia at a rate *σγ*, *σ* > 1. If the miracidium reaches a fresh water with snails of a suitable species, it penetrates at a rate *λ*_*M*_, where

(4) $$\begin{array}{c} \begin{array}{c} \lambda_{M}=\frac{\beta_{M}M\left(t\right)}{M_{0}+\epsilon M\left(t\right)}, \end{array} \end{array}$$

and transforms into a sporocyst otherwise, the miracidia die naturally at a rate *μ*_*M*_. The infected snails release a second form of free swimming larva called a cercariae which is capable of infecting humans at rate *θ*. Some cercariae also die naturally at a rate *μ*_*P*_. Individuals infected with schistosomiasis are infected with HIV at a rate *δλ*_*H*_ with *δ* > 1 since infection by schistosomiasis creates wounds within the urethra as eggs are being released, which increases the likelihood of HIV infection per sexual contact. Individuals with HIV progress to the AIDS stage at a rate *ρ*. Individuals in the AIDS stage have an additional disease-induced death rate *d*_*A*_. We assume that antiretroviral therapy is given to AIDS individuals who are ill and have experienced AIDS-defining symptoms, or whose CD4+ T cell count is below 200/*μ*L, which is the recommended AIDS defining stage [42]. Thus, AIDS patients are assumed to get antiretroviral therapy at a constant rate *α*. Treated AIDS patients eventually succumb to AIDS-induced mortality at a reduced rate modeled by the parameter *τ* (0 < *τ* < 1). Individuals treated for schistosomiasis are assumed to recover at a constant rate *ω*, and *ω*_1_ denotes AIDS patients who have recovered from schistosomiasis but are on antiretroviral therapy since the latter is a life treatment. The model flowchart for the interaction of the two diseases is shown in Figure 1 and parameters described will assume values in Table 1.

From the aforementioned model description and assumptions, we establish the following deterministic system of nonlinear differential equations

(5) $$\begin{array}{c} \text{Model}\;\text{system}\{\begin{array}{c} \frac{dS_{H}}{dt}=\Lambda_{H}+\omega I_{B}-\left(\lambda_{H}+\lambda_{P}\right)S_{H}-\mu_{H}S_{H},\\ \frac{dI_{B}}{dt}=\lambda_{P}S_{H}-\delta \lambda_{H}I_{B}-\left(\mu_{H}+\omega +d_{B}\right)I_{B},\\ \frac{dI_{H}}{dt}=\lambda_{H}S_{H}+\omega I_{H_{B}}-\lambda_{P}I_{H}-\left(\mu_{H}+\rho \right)I_{H},\\ \frac{dA_{H}}{dt}=\rho I_{H}+\omega A_{H_{B}}-\lambda_{P}A_{H}-\left(\mu_{H}+\alpha +d_{A}\right)A_{H},\\ \frac{dA_{H_{T_{A}}}}{dt}=\alpha A_{H}+\omega A_{H_{T_{B}}}-\lambda_{p}A_{H_{T_{A}}}\\ \;\;\;\;-\left(\mu_{H}+\tau d_{A}\right)A_{H_{T_{A}}},\\ \frac{dI_{H_{B}}}{dt}=\delta \lambda_{H}I_{B}+\lambda_{P}I_{H}-\left(\rho +\omega +\mu_{H}+d_{B}\right)I_{H_{B}},\\ \frac{dA_{H_{B}}}{dt}=\lambda_{P}A_{H}+\rho I_{H_{B}}-\left(\mu_{H}+\omega +d_{A}+d_{B}\right)A_{H_{B}},\\ \frac{dA_{H_{T_{B}}}}{dt}=\lambda_{P}A_{H_{T_{A}}}+\alpha A_{H_{B}}\\ \;\;\;\;-\left(\mu_{H}+\omega +\tau d_{A}+d_{B}\right)A_{H_{T_{B}}},\\ \frac{dM}{dt}=N_{E}\gamma \left(I_{B}+I_{H_{B}}+\sigma A_{H_{B}}+\sigma A_{H_{T_{B}}}\right)-\mu_{M}M,\\ \frac{dS_{S}}{dt}=\Lambda_{S}-\lambda_{M}S_{S}-\mu_{S}S_{S},\\ \frac{dI_{S}}{dt}=\lambda_{M}S_{S}-\left(\mu_{S}+d_{S}\right)I_{S},\\ \frac{dP}{dt}=\theta I_{S}-\mu_{P}P. \end{array} \end{array}$$

### 2.1. Model Basic Properties

In this section, we study the basic properties of the solutions of model system (5), which are essential in the proofs of stability.

**Lemma 1**

The equations preserve positivity of solutions.

**Proof**

Considering the human population only, the vector field given by the right-hand side of (5) points inward on the boundary of ℝ_+_^8^∖{0}. For example, if *A*_*H*_ = 0, then, *A*_*H*_′ = *ρI*_*H*_ + *ωA*_*H*_*B*__ ≥ 0. In an analogous manner, the same result can be shown for the other model components (variables). We shall use the human population to illustrate the boundedness of solutions for model system (5).

**Lemma 2**

Each nonnegative solution of model system (5) is bounded in *L* ^1^-norm.

**Proof**

Consider the human population only, and let *L*_*H*_^1^ ∈ *L* ^1^; then, the norm *L*_*H*_^1^ of each nonnegative solution in *N*_*H*_ is given by max {*N*_*H*_(0), Λ_*H*_/*μ*_*H*_}. Thus, the norm *L*_*H*_^1^ satisfies the inequality *N*_*H*_′ ≤ Λ − *μ*_*H*_ *N*_*H*_. Solutions to the equation *Q*′ = Λ − *μQ* are monotone increasing and bounded by Λ/*μ* if *Q*(0) < Λ/*μ*. They are monotone decreasing and bounded above if *Q*(0) ≥ Λ/*μ*. Since *N*_*H*_′ ≤ *Q*′, the claim follows and in a similar fashion, the remaining model variables can be shown to bounded.

**Corollary 1**

The region

(6) $$\begin{array}{c} \Phi =\{\begin{array}{c} \left(S_{H},I_{B},I_{H},A_{H},A_{H_{T_{A}}},I_{H_{B}},A_{H_{B}},A_{H_{T_{B}}}\right)\\ \;\in {\mathbb{R}}_{+}^{8}:N_{H}\leq \frac{\Lambda_{H}}{\mu_{H}},\\ M\in {\mathbb{R}}_{+}:M\leq \frac{\gamma \Lambda_{H}N_{E}\left(1+\sigma \right)}{\mu_{M}\mu_{H}},\\ \left(S_{S},I_{S}\right)\in {\mathbb{R}}_{+}^{2}:N_{S}\leq \frac{\Lambda_{S}}{\mu_{S}},\\ P\in {\mathbb{R}}_{+}:P\leq \frac{\theta \Lambda_{S}}{\mu_{P}\mu_{S}} \end{array} \end{array}$$

is invariant and attracting for system (5).

**Theorem 1**

For every nonzero, nonnegative initial value, solutions of model system (5) exist for all time *t* > 0.

**Proof**

Local existence of solutions follows from standard arguments since the right-hand side of (5) is locally Lipschitz. Global existence follows from the* a priori* bounds.

## 3. Analysis of the Submodels

Before analyzing the full model system (5), it is essential to gain insights into the dynamics of the models for HIV only and schistosomiasis only.

### 3.1. HIV-Only Model

We now consider a model for HIV/AIDS only, obtained by setting *I*_*B*_ = *I*_*H*_*B*__ = *A*_*H*_*B*__ = *A*_*H*_*T*_*B*___ = *M* = *S*_*S*_ = *I*_*S*_ = *P* = 0, so that system in (5) reduces to

(7) $$\begin{array}{c} \text{HIV}/\text{AIDS}\;\text{only}\;\{\begin{array}{c} \frac{dS_{H}}{dt}=\Lambda_{H}-\left(\lambda_{H}+\mu_{H}\right)S_{H},\\ \frac{dI_{H}}{dt}=\lambda_{H}S_{H}-\left(\rho +\mu_{H}\right)I_{H},\\ \frac{dA_{H}}{dt}=\rho I_{H}-\left(\alpha +d_{A}+\mu_{H}\right)A_{H},\\ \frac{dA_{H_{T_{A}}}}{dt}=\alpha A_{H}-\left(\tau d_{A}+\mu_{H}\right)A_{H_{T_{A}}},\\ \text{with},\;\lambda_{H}=\frac{\beta_{H}c\left[I_{H}+\eta A_{H}+\kappa A_{H_{T_{A}}}\right]}{N_{H}},\\ \;\;\;N_{H}=S_{H}+I_{H}+A_{H}+A_{H_{T_{A}}}. \end{array} \end{array}$$

For system (7), it can be shown that the region

(8) $$\begin{array}{c} \Phi_{H}=\left\{\left(S_{H},I_{H},A_{H},A_{H_{T_{A}}}\right)\in {\mathbb{R}}_{+}^{4}:N_{H}\leq \frac{\Lambda_{H}}{\mu_{H}}\right\} \end{array}$$

is invariant and attracting. Thus, the dynamics of the HIV-only model will be considered in Φ_*H*_.

#### 3.1.1. Disease-Free Equilibrium and Stability Analysis

Model system (7) has an evident disease-free given by

(9) $${𝒰}_{0H}=\left(S_{H}^{0},I_{H}^{0},A_{H}^{0},A_{H_{T_{A}}}^{0}\right)=\left(\frac{\Lambda_{H}}{\mu_{H}},0,0,0\right).$$

Following the next generation approach and the notation defined therein [43], matrices *F* and *V* for new infection terms and the remaining transfer terms are, respectively, given by

(10) $$\begin{array}{c} F=\left[\begin{array}{ccc} \beta_{H}c & \beta_{H}c\eta & \beta_{H}c\kappa \\ 0 & 0 & 0\\ 0 & 0 & 0 \end{array}\right],\\ V=\left[\begin{array}{ccc} \mu_{\text{H}} & 0 & 0\\ 0 & \mu_{H}+\rho & 0\\ 0 & 0 & \mu_{H}+\tau d_{A} \end{array}\right]. \end{array}$$

It follows from (10) that the reproduction number of the system (7) is given by

(11) $$\begin{array}{c} {ℛ}_{A}=\frac{\beta_{H}c\left[\rho \kappa \alpha +\left(\mu_{H}+\tau d_{A}\right)\left(\eta \rho +\alpha +\mu_{H}+d_{A}\right)\right]}{\left(\mu_{H}+\rho \right)\left(\mu_{H}+d_{A}\right)\left(\mu_{H}+\alpha +d_{A}\right)}. \end{array}$$

The threshold quantity *ℛ*_*A*_ measures the average number of new secondary cases generated by a single individual in a population where the aforementioned HIV control measures are in place. An associated epidemiological threshold which is the* basic reproductive number ℛ*_0_, obtained using the same technique of the next generation operator [43], by considering model system (7) in the absence of HIV intervention strategies, is given by

(12) $$\begin{array}{c} {ℛ}_{0_{A}}=\frac{\beta_{H}c\left(\mu_{H}+d_{A}+\eta \rho \right)}{\left(\mu_{H}+\rho \right)\left(\mu_{H}+d_{A}\right)}. \end{array}$$

This disease threshold quantity *ℛ*_0_*A*__ measures the average number of new infections generated by a single infected individual in a completely susceptible population where there are no HIV intervention strategies. Using Theorem 2 in [43], the following result is established.

**Lemma 3**

The disease-free equilibrium *𝒰*_0*H*_ of system (7) is locally asymptotically stable (LAS) if *ℛ*_*A*_ < 1 and unstable if *ℛ*_*A*_ > 1.

#### 3.1.2. Sensitivity Analysis of HIV-Only-Induced Reproductive Number

To avoid repetition we refer the reader to a detailed analysis of the reproductive number for model system (7), in the work of Bhunu et al. [44].

#### 3.1.3. Global Stability of HIV/AIDS Model

We claim the following result.

**Lemma 4**

The disease-free equilibrium (*𝒰*_0*H*_) of model system (7) is globally asymptotically stable (GAS) if *ℛ*_*A*_ < 1 and unstable if *ℛ*_*A*_ > 1.

**Proof**

The proof is based on using a comparison theorem [45]. Note that the equations of the infected components in system (7) can be written as

(13) $$\begin{array}{cc} \left[\begin{array}{c} \frac{dI_{H}}{dt}\\ \frac{dA_{H}}{dt}\\ \frac{dA_{H_{T_{A}}}}{dt} \end{array}\right] & =\left[F-V\right]\left[\begin{array}{c} I_{H}\\ A_{H}\\ A_{H_{T_{A}}} \end{array}\right]\\ & \;-\beta_{H}c\left[1-\frac{S_{H}}{N_{H}}\right]\left[\begin{array}{ccc} 1 & \eta & \kappa \\ 0 & 0 & 0\\ 0 & 0 & 0 \end{array}\right]\left[\begin{array}{c} I_{H}\\ A_{H}\\ A_{H_{T_{A}}} \end{array}\right], \end{array}$$

where *F* and *V*, are as defined earlier in (10). Since *S*_*H*_ ≤ *N*_*H*_, (for all *t* ≥ 0) in Φ_*H*_, it follows that

(14) $$\left[\begin{array}{c} \frac{dI_{H}}{dt}\\ \frac{dA_{H}}{dt}\\ \frac{dA_{H_{T_{A}}}}{dt} \end{array}\right]\leq \left[F-V\right]\left[\begin{array}{c} I_{H}\\ A_{H}\\ A_{H_{T_{A}}} \end{array}\right].$$

Using the fact that the eigenvalues of the matrix *F* − *V* all have negative real parts, it follows that the linearized differential inequality system (14) is stable whenever *ℛ*_*A*_ < 1. Consequently, (*I*_*H*_, *A*_*H*_, *A*_*H*_*T*_*A*___)→(0,0, 0) as *t* → *∞*. Thus, by a comparison theorem [45] (*I*_*H*_, *A*_*H*_, *A*_*H*_*T*_*A*___)→(0,0, 0) as *t* → *∞*, and evaluating system (7) at *I*_H_ = *A*_*H*_ = *A*_*H*_*T*_*A*___ = 0 gives *S*_*H*_ → *S*_*H*_^0^ for *ℛ*_*A*_ < 1. Hence, the DFE (*𝒰*_0*H*_) is GAS for *ℛ*_*A*_ < 1.

#### 3.1.4. HIV-Only Equilibrium

Expressed in terms of the equilibrium value of the force of infection *λ*_H_*, this equilibrium is given by

(15) $${𝒰}_{1}^{*}\{\begin{array}{c} S_{H}^{*}=\frac{\Lambda_{H}}{\mu_{H}+\lambda_{H}^{*}},\\ I_{H}^{*}=\frac{\Lambda_{H}\lambda_{H}^{*}}{\left(\mu_{H}+\lambda_{H}^{*}\right)\left(\mu_{H}+\rho \right)},\\ A_{H}^{*}=\frac{\rho \lambda_{H}^{*}\Lambda_{H}}{\left(\mu_{H}+\lambda_{H}^{*}\right)\left(\mu_{H}+\rho \right)\left(\mu_{H}+\alpha +d_{A}\right)},\\ A_{H_{T_{A}}}^{*}=\frac{\alpha \rho \lambda_{H}^{*}\Lambda_{H}}{\left(\mu_{H}+\lambda_{H}^{*}\right)\left(\mu_{H}+d_{A}\right)\left(\mu_{H}+\rho \right)\left(\mu_{H}+\alpha +d_{A}\right)}. \end{array}$$

The local bifurcation analysis is based on the centre manifold approach [46] as described by Theorem 4.1 in [47], stated in the appendix for convenience (also see [43] for more details). To apply the said Theorem 10 in order to establish the local asymptotic stability of the endemic equilibrium, it is convenient to make the following change of variables: *S*_*H*_ = *x*_1_, *I*_*H*_ = *x*_2_, *A*_*H*_ = *x*_3_, and *A*_*H*_*T*_*A*___ = *x*_4_, so that *N*_*H*_ = ∑_*n*=1_^4^ *x*_*n*_. We now use the vector notation *X* = (*x*_1_, *x*_2_, *x*_3_, *x*_4_)^*T*^. Then, model system (7) can be written in the form *dX*/*dt* = *F* = (*f*_1_, *f*_2_, *f*_3_, *f*_4_)^*T*^, where

(16) $$\begin{array}{cc} x_{1}^{\prime }\left(t\right) & =f_{1}=\Lambda_{H}-\frac{\beta_{H}c\left(x_{2}+\eta x_{3}+\kappa x_{4}\right)}{\sum_{n=1}^{4}x_{n}}x_{1}-\mu_{H}x_{1},\\ x_{2}^{\prime }\left(t\right) & =f_{2}=\frac{\beta_{H}c\left(x_{2}+\eta x_{3}+\kappa x_{4}\right)}{\sum_{n=1}^{4}x_{n}}x_{1}-\left(\mu_{H}+\rho \right)x_{2},\\ x_{3}^{\prime }\left(t\right) & =f_{3}=\rho x_{2}-\left(\mu_{H}+\alpha +d_{A}\right)x_{3},\\ x_{4}^{\prime }\left(t\right) & =f_{4}=\alpha x_{3}-\left(\mu_{H}+\tau d_{A}\right)x_{4}. \end{array}$$

The Jacobian matrix of system (16) at *𝒰*_0_ is given by

(17) $$\begin{array}{cc} & J\left({𝒰}_{0H}\right)\\ & =\left[\begin{array}{cccc} -\mu_{H} & -\beta_{H}c & -\eta \beta_{H}c & -\kappa \beta_{H}c\\ 0 & \beta_{H}c-\left(\mu_{H}+\rho \right) & \eta \beta_{H}c & \kappa \beta_{H}c\\ 0 & \rho & -\left(\mu_{H}+\alpha +d_{A}\right) & 0\\ 0 & 0 & \alpha & -\left(\mu_{H}+\tau d_{A}\right) \end{array}\right], \end{array}$$

from which it can be shown that the HIV/AIDS-induced reproduction number is

(18) $${ℛ}_{A}=\frac{\beta_{H}c\left[\kappa \rho \alpha +\left(\mu_{H}+\tau d_{A}\right)\left(\eta \rho +\alpha +\mu_{H}+d_{A}\right)\right]}{\left(\mu_{H}+\rho \right)\left(\mu_{H}+d_{A}\right)\left(\mu_{H}+\alpha +d_{A}\right)}.\;$$

If *β*_*H*_ is taken as a bifurcation parameter and by solving for *β*_*H*_ when *ℛ*_*A*_ = 1, we obtain

(19) $$\beta_{H}=\beta_{H}^{*}=\frac{\left(\mu_{H}+\rho \right)\left(\mu_{H}+d_{A}\right)\left(\mu_{H}+\alpha +d_{A}\right)}{c\left[\kappa \rho \alpha +\left(\mu_{H}+\tau d_{A}\right)\left(\eta \rho +\alpha +\mu_{H}+d_{A}\right)\right]}.\;$$

Note that the linearized system of the transformed model (16) with *β*_*H*_ = *β*_H_* has a simple zero eigenvalue, which allows the use of Castillo-Chavez and Song result [47] to analyze the dynamics of (16) near *β*_*H*_ = *β*_H_*. It can be shown that the Jacobian of (16) at *β*_*H*_ = *β*_H_* has a right eigenvector associated with the zero eigenvalue given by *u* = [*u*_1_, *u*_2_, *u*_3_, *u*_4_]^*T*^, where

(20) $$\begin{array}{cc} & u_{1}=\frac{-\beta_{H}c\left(u_{2}+\eta u_{3}+\kappa u_{4}\right)}{\mu_{H}},\;u_{2}>0,\\ & u_{3}=\frac{\rho u_{2}}{\alpha +d_{A}+\mu_{H}},\;u_{4}=\frac{\alpha u_{3}}{\mu_{H}+\tau d_{A}}. \end{array}$$

The left eigenvector of *J*(*𝒰*_0*H*_) associated with the zero eigenvalue at *β*_*H*_ = *β*_H_* is given by *v* = [*v*_1_, *v*_2_, *v*_3_, *v*_4_]^*T*^, where

(21) $$v_{1}=0,\;v_{2}=\frac{\rho v_{3}}{\mu_{H}+\rho -\beta_{H}^{*}c},\;v_{3}>0,\;v_{4}=\frac{\kappa \beta_{H}cv_{2}}{\mu_{H}+\tau d_{A}}.$$

**Computation of the Bifurcation Parameters *a* and *b***

The application of Theorem 10 (see the appendix) entails the computation of two parameters *a* and *b*, say. After some little algebraic manipulations and rearrangements, it can be shown that

(22) $$a=-\frac{2\beta_{H}^{*}c\mu_{H}v_{2}}{\Lambda_{H}}\left(u_{2}+u_{3}+u_{4}\right)\left(u_{2}+\eta u_{3}+\kappa u_{4}\right)<0.\;$$

Furthermore,

(23) $$b=c\left(u_{2}+\eta u_{3}+\kappa u_{4}\right)v_{2}>0.$$

This sign of *b* may be expected in general for epidemic models because, in essence, using *β* as a bifurcation parameter often ensures *b* > 0 [43]. Since *a* < 0 (which excludes any possibility of multiple equilibria and hence backward bifurcation), model system (16) has a forward (or transcritical) bifurcation at *ℛ*_*A*_ = 1, and consequently, the local stability implies global stability. This result is summarized below.

**Theorem 2**

The endemic equilibrium *𝒰*_1_* is locally asymptotically stable for *ℛ*_*A*_ > 1.

### 3.2. Schistosomiasis-Only Model

In the absence of HIV/AIDS in the community (obtained by setting HIV/AIDS-related parameters to zero from system (5)) schistosomiasis-only model is given by

(24) $$\begin{array}{c} \text{Schistosomiasis-only}\;\text{model}\{\begin{array}{c} \frac{dS_{H}}{dt}=\Lambda_{H}+\omega I_{B}-\left(\lambda_{P}+\mu_{H}\right)S_{H},\\ \frac{dI_{B}}{dt}=\lambda_{H}I_{B}-\left(\mu_{H}+\omega +d_{B}\right)I_{B},\\ \frac{dM}{dt}=N_{E}\gamma I_{B}-\mu_{M}M,\\ \frac{dS_{S}}{dt}=\Lambda_{S}-\lambda_{M}S_{S}-\mu_{S}S_{S},\\ \frac{dI_{S}}{dt}=\lambda_{M}S_{S}-\left(\mu_{S}+d_{S}\right)I_{S},\\ \frac{dP}{dt}=\theta I_{S}-\mu_{P}P,\\ \text{with},\;\lambda_{P}=\frac{\beta_{P}P\left(t\right)}{P_{0}+\epsilon P\left(t\right)},\\ \;\;\;\;\lambda_{M}=\frac{\beta_{M}M\left(t\right)}{M_{0}+\epsilon M\left(t\right)}. \end{array} \end{array}$$

For system (24), it can be shown that the region

(25) $$\Phi_{B}=\{\begin{array}{c} \left(S_{H},I_{B}\right)\in {\mathbb{R}}_{+}^{2}:N_{H}\leq \frac{\Lambda_{H}}{\mu_{H}},\\ M\in {\mathbb{R}}_{+}:M\leq \frac{\gamma \Lambda_{H}N_{E}\left(1+\sigma \right)}{\mu_{M}\mu_{H}},\\ \left(S_{S},I_{S}\right)\in {\mathbb{R}}_{+}^{2}:N_{S}\leq \frac{\Lambda_{S}}{\mu_{S}},\\ P\in {\mathbb{R}}_{+}:P\leq \frac{\theta \Lambda_{S}}{\mu_{P}\mu_{S}} \end{array}$$

is invariant and attracting. Thus, the dynamics of schistosomiasis-only model will be considered in Φ_*B*_.

#### 3.2.1. Disease-Free Equilibrium and Stability Analysis

Model system (24) has an evident disease-free given by

(26) $${𝒰}_{0B}=\left(S_{H}^{0},I_{B}^{0},M^{0},S_{S}^{0},I_{S}^{0},P^{0}\right)=\left(\frac{\Lambda_{H}}{\mu_{H}},0,0,\frac{\Lambda_{S}}{\mu_{S}},0,0\right).\;$$

Following van den Driessche and Watmough [43], the reproduction number of the model system (24) is given by

(27) $$\begin{array}{cc} {ℛ}_{B} & =\sqrt{\left(\frac{\beta_{p}N_{E}\gamma \Lambda_{H}}{\mu_{M}\mu_{H}P_{0}\left(\mu_{H}+\omega +d_{B}\right)}\right)\left(\frac{\beta_{M}\theta \Lambda_{S}}{\mu_{P}\mu_{S}M_{0}\left(\mu_{S}+d_{S}\right)}\right)}\\ & =\sqrt{{ℛ}_{H}{ℛ}_{S}} \end{array}$$

where *ℛ*_*H*_ = *β*_*p*_ *N*_*E*_ *γ*Λ_*H*_/*μ*_*M*_ *μ*_*H*_ *P*_0_(*μ*_*H*_ + *ω* + *d*_*B*_) represents the snail-man initial disease transmission and *ℛ*_*S*_ = *β*_*M*_ *θ*Λ_*S*_/*μ*_*P*_ *μ*_*S*_ *M*_0_(*μ*_*S*_ + *d*_*S*_) is the man-snail initial disease transmission. 

The threshold quantity *ℛ*_*B*_ measures the average number of new secondary cases generated by a single individual in a population where there is schistosomiasis treatment. An associated epidemiological threshold, *ℛ*_0_*B*__, obtained using a similar technique of the next generation by considering model system (24) in the absence of schistosomiasis treatment is given by

(28) $$\begin{array}{cc} {ℛ}_{0_{B}} & =\sqrt{\left(\frac{\beta_{p}N_{E}\gamma \Lambda_{H}}{\mu_{M}\mu_{H}P_{0}\left(\mu_{H}+d_{B}\right)}\right)\left(\frac{\beta_{M}\theta \Lambda_{S}}{\mu_{P}\mu_{\text{S}}M_{0}\left(\mu_{S}+d_{S}\right)}\right)}\\ & =\sqrt{{ℛ}_{0_{H}}{ℛ}_{0_{S}}}, \end{array}$$

where *ℛ*_0_*H*__ = *β*_*p*_ *N*_*E*_ *γ*Λ_*H*_/*μ*_*M*_ *μ*_*H*_ *P*_0_(*μ*_*H*_ + *d*_*B*_) represents the snail-man initial disease transmission and *ℛ*_0_*S*__ = *β*_*M*_ *θ*Λ_*S*_/*μ*_*P*_ *μ*_*S*_ *M*_0_(*μ*_*S*_ + *d*_*S*_) is the man-snail initial disease transmission. Using Theorem 2 in [43], the following result is established.

**Theorem 3**

The disease-free equilibrium *𝒰*_0_*B*__ is locally asymptotically stable whenever *ℛ*_*B*_ < 1 and unstable otherwise.

**Impact of Schistosomiasis Treatment in the Community**

Here, the reproductive number *ℛ*_*B*_ is analyzed to determine whether or not treatment of schistosomiasis patients (modeled by the rate *ω*) can lead to the effective control of schistosomiasis in the community. It follows from (27) that the elasticity [48] of *ℛ*_*B*_ with respect to *ω* can be computed using the approach in [49] as follows:

(29) $$\frac{\omega }{{ℛ}_{B}}\frac{\partial {ℛ}_{B}}{\partial \omega }=-\frac{\omega }{2\left(\mu_{H}+\omega +d_{B}\right)}<0.$$

The sensitivity index of the reproduction number is used to assess the impact on the relevant parameters to disease transmission. That is, the elasticity measures the effect a change in *ω*, say, has as a proportional change in *ℛ*_*B*_, and from (29), we note that an increase in *ω* will lead to a decrease in *ℛ*_*B*_, thus (29) suggests that an increase in treatment of schistosomiasis patients does have a positive impact in controlling schistosomiasis in the community (assuming full compliance to the therapy, no treatment failure, and no development of resistance).

### 3.3. Global Stability of the Disease-Free Equilibrium

We shall use the following theorem of Castillo-Chavez et al. [50] in the sequel.

**Theorem 4 (see [50])**

If system (5) can be written in the form

(30) $$\begin{array}{c} \frac{dX}{dt}=F\left(x,Z\right),\\ \frac{dZ}{dt}=G\left(X,Z\right),\;G\left(x,0\right)=0, \end{array}$$

where *X* ∈ ℝ^*m*^ denotes (its components) the number of uninfected individuals, *Z* ∈ ℝ^*n*^ denotes (its components) the number of infected individuals including latent and infectious, and **U**_0_ = (x*, 0) denotes the disease-free equilibrium of the system. Assume that (i) for *dX*/*dt* = *F*(*X*, 0), X* is globally asymptotically stable, (ii) $G(X,Z)=AZ-\hat{G}(X,Z)$, $\hat{G}(X,Z)\geq 0$ for (*X*, *Z*) ∈ *𝒟*, where *A* = *D*_*Z*_ *G*(X*, 0) is an *M*-matrix (the off-diagonal elements of *A* are nonnegative) and *𝒟* is the region where the model makes biological sense. Then the fixed point **U**_0_ = (x*, 0) is a globally asymptotic stable equilibrium of model system (5) provided *ℛ*_*B*_ < 1.

Applying Theorem 4 to model system (5) yields

(31) $$\begin{array}{cc} \hat{G}\left(X,Y\right) & =\left[\begin{array}{c} \hat{G_{1}}\left(X,Y\right)\\ \hat{G_{2}}\left(X,Y\right)\\ \hat{G_{3}}\left(X,Y\right)\\ \hat{G_{4}}\left(X,Y\right) \end{array}\right]=\left[\begin{array}{c} \beta_{P}P\left(\frac{\Lambda_{H}}{P_{0}\mu_{H}}-\frac{S_{H}}{P_{0}+\epsilon P}\right)\\ \beta_{M}M\left(\frac{\Lambda_{S}}{M_{0}\mu_{S}}-\frac{S_{S}}{M_{0}+\epsilon M}\right)\\ 0\\ 0 \end{array}\right]. \end{array}$$

Since *S*_*H*_^0^( = Λ_*H*_/*μ*_*H*_)(1/*P*_0_) ≥ *S*_*H*_/(*P*_0_ + *ϵP*) and *S*_*S*_( = Λ_*S*_/*μ*_*S*_)(1/*M*_0_) ≥ *S*_*S*_/(*M*_0_ + *ϵM*), it follows that $\hat{G}(X,Y)\geq 0$. We summarise the result in Theorem 5.

**Theorem 5**

The disease-free equilibrium (*𝒰*_0*B*_) of model system (24) is globally asymptotically stable (GAS) if *ℛ*_*B*_ < 1 and unstable if *ℛ*_*B*_ > 1.

#### 3.3.1. Schistosomiasis-Only Equilibrium

Model system (24) has an endemic equilibrium denoted by *𝒰*_2_*, where

(32) $${𝒰}_{2}^{*}\{\begin{array}{c} S_{H}^{**}=\frac{\Lambda_{H}}{\mu_{H}+\omega +\lambda_{P}^{**}},\\ I_{B}^{**}=\frac{\Lambda_{H}\lambda_{P}^{**}}{\left(\mu_{H}+\omega +\lambda_{P}^{**}\right)\left(\mu_{H}+\omega +d_{B}\right)},\\ M^{**}=\frac{N_{E}\gamma \Lambda_{H}\lambda_{P}^{**}}{\mu_{M}\left(\mu_{H}+\omega +\lambda_{P}^{**}\right)\left(\mu_{H}+\omega +d_{B}\right)},\\ S_{S}^{**}=\frac{\Lambda_{S}}{\mu_{S}+\lambda_{S}^{**}},\\ I_{S}^{**}=\frac{\Lambda_{S}\lambda_{M}^{**}}{\left(\mu_{S}+\lambda_{M}^{**}\right)\left(\mu_{S}+d_{S}\right)},\\ P^{**}=\frac{\theta \Lambda_{S}\lambda_{S}^{**}}{\mu_{P}\left(\mu_{S}+\lambda_{S}^{**}\right)\left(\mu_{S}+d_{S}\right)},\\ \text{with}\;\lambda_{P}^{**}=\frac{\beta_{P}P^{**}}{P_{0}+\epsilon P^{**}},\;\lambda_{M}^{**}=\frac{\beta_{M}M^{**}}{M_{0}+\epsilon M^{**}}. \end{array}$$

The local asymptotic stability of the endemic equilibrium *𝒰*_2_* can also be analyzed using the centre manifold theory. In this case, the Jacobian matrix of the system at *𝒰*_0*B*_ is given by

(33) $$\begin{array}{cc} & J\left({𝒰}_{0B}\right)\\ & =\left[\begin{array}{cccccc} -\mu_{H} & 0 & 0 & 0 & 0 & -\frac{\beta_{P}\Lambda_{H}}{P_{0}\mu_{H}}\\ 0 & -\left(\mu_{H}+\omega +d_{B}\right) & 0 & 0 & 0 & \frac{\beta_{P}\Lambda_{H}}{P_{0}\mu_{H}}\\ 0 & N_{E}\gamma & -\mu_{M} & 0 & 0 & 0\\ 0 & 0 & -\frac{\beta_{M}\Lambda_{S}}{M_{0}\mu_{S}} & -\mu_{S} & 0 & 0\\ 0 & 0 & \frac{\beta_{M}\Lambda_{S}}{M_{0}\mu_{S}} & 0 & -\left(\mu_{S}+d_{S}\right) & 0\\ 0 & 0 & 0 & 0 & \theta & -\mu_{P} \end{array}\right]. \end{array}$$

If *β*_*P*_ is taken as a bifurcation parameter, and solving for *β*_*P*_ when *ℛ*_*B*_ = 1, we obtain

(34) $$\beta_{P}=\beta_{P}^{*}=\frac{\mu_{M}\mu_{H}P_{0}\left(\mu_{H}+\omega +d_{B}\right)}{N_{E}\gamma \Lambda_{H}{ℛ}_{S}}.$$

The linearized system of the the model with *β*_*P*_ = *β*_P_* has a simple zero eigenvalue. Therefore, it can be shown that the above Jacobian has a right eigenvector given by *w* = [*w*_1_, *w*_2_, *w*_3_, *w*_4_, *w*_5_, *w*_6_]^*T*^, where

(35) $$\begin{array}{c} \begin{array}{c} w_{1}=-\frac{\beta_{P}^{*}\Lambda_{H}{ℛ}_{0_{S}}w_{3}}{P_{0}\mu_{H}^{2}},\;w_{2}=\frac{\mu_{P}\beta_{P}^{*}\Lambda_{H}w_{3}}{\theta \left(\mu_{H}+\omega +d_{B}\right)},\;w_{3}=w_{3},\\ w_{4}=-\frac{\beta_{M}\Lambda_{S}w_{3}}{M_{0}\mu_{S}^{2}},\;w_{5}=\frac{\beta_{M}\Lambda_{S}w_{3}}{\left(\mu_{S}+d_{S}\right)M_{0}\mu_{S}},\;w_{6}={ℛ}_{0_{S}}w_{3}. \end{array} \end{array}$$

The left eigenvector of *J*(*𝒰*_0_*B*__) associated with the zero eigenvalue at *β*_*P*_ = *β*_P_* is given by *z* = [*z*_1_, *z*_2_, *z*_3_, *z*_4_, *z*_5_, *z*_6_]^*T*^, where

(36) $$\begin{array}{c} z_{1}=0=z_{4},\;z_{3}>0,\;z_{2}=\frac{N_{E}\gamma z_{3}}{\mu_{H}+\omega +d_{B}},\\ \begin{array}{c} z_{5}=\frac{\mu_{M}M_{0}\mu_{S}z_{3}}{\beta_{M}\Lambda_{S}},\;z_{6}=\frac{\beta_{P}^{*}\Lambda_{H}N_{E}\gamma z_{3}}{P_{0}\mu_{H}\mu_{P}\left(\mu_{H}+\omega +d_{B}\right)}. \end{array} \end{array}$$

Computation of the bifurcation coefficients *a* and *b* yields

(37) $$\begin{array}{cc} a & =-2z_{3}w_{3}^{2}(\frac{N_{E}\gamma {ℛ}_{S}^{2}\beta_{P}^{*}\Lambda_{H}\left(\beta_{P}^{*}+\epsilon \mu_{H}\right)}{\left(\mu_{H}+\omega +d_{B}\right)P_{0}^{2}\mu_{H}^{2}}\\ & \;\;\;\;\;+\frac{N_{E}\gamma \theta \beta_{P}^{*}\Lambda_{H}\beta_{M}\Lambda_{S}\left(\beta_{M}+\epsilon \mu_{S}\right)}{\left(\mu_{H}+\omega +d_{B}\right)\left(\mu_{S}+d_{S}\right)P_{0}\mu_{H}M_{0}^{2}\mu_{S}^{2}})<0,\\ b & =\frac{N_{E}\gamma {ℛ}_{S}\Lambda_{S}z_{3}w_{3}}{\left(\mu_{H}+\omega +d_{B}\right)P_{0}\mu_{S}}>0. \end{array}$$

Thus, the following result is established.

**Theorem 6**

The unique endemic equilibrium *𝒰*_2_* is locally asymptotically stable for *ℛ*_*B*_ > 1.

Since *a* < 0, local stability of *𝒰*_2_* implies its global stability.

## 4. HIV/AIDS and Schistosomiasis Model

Model system (5) has evident disease-free (DFE) given by

(38) $$\begin{array}{cc} {𝒰}_{0} & =\left(S_{H}^{0},I_{B}^{0},I_{H}^{0},A_{H}^{0},A_{H_{T_{A}}}^{0},I_{H_{B}}^{0},A_{H_{B}}^{0},A_{H_{T_{B}}}^{0},M^{0},S_{S}^{0},I_{S}^{0},P^{0}\right)\\ & =\left(\frac{\Lambda_{H}}{\mu_{H}},0,0,0,0,0,0,0,0,\frac{\Lambda_{S}}{\mu_{S}},0,0\right). \end{array}$$

Following van den Driessche and Watmough [43], the reproduction number of the model is

(39) $${ℛ}_{H_{B}}=\max\;\left\{{ℛ}_{A},{ℛ}_{B}\right\}$$

with *ℛ*_*A*_ and *ℛ*_*B*_ defined as earlier in Section 3 above. Using Theorem 2 in [43], the following result is established.

**Theorem 7**

The disease-free equilibrium *𝒰*_0_ is locally asymptotically stable whenever *ℛ*_*H*_*B*__ < 1 and unstable otherwise.

### 4.1. Sensitivity Analysis

In this section we investigate the effects of HIV/AIDS on schistosomiasis and vice versa, in the presence and absence of the aforementioned intervention strategies.

**Impact of Schistosomiasis on HIV/AIDS in the Absence of Control Measures**

To analyze the effects of schistosomiasis on HIV/AIDS and vice versa in the absence of control measures for either HIV/AIDS or schistosomiasis, we begin by introducing the following notation; in the absence of antiretroviral therapy (*α* = 0) the reproductive number is denoted by *ℛ*_0_*A*__ and also in the absence of schistosomiasis treatment (*ω* = 0), *ℛ*_*B*_ = *ℛ*_0_*B*__. Thus, to express *ℛ*_0_*B*__ in terms of *ℛ*_0_*A*__, we solve for *μ*_*H*_ and obtain

(40) $$\begin{array}{c} \mu_{H}=\frac{-\left(\phi_{1}{ℛ}_{0_{A}}+\phi_{2}\right)+\sqrt{\phi_{3}{ℛ}_{0_{A}}^{2}+\phi_{4}{ℛ}_{0_{A}}+\phi_{5}}}{2{ℛ}_{0_{A}}}, \end{array}$$

where

(41) $$\begin{array}{cc} & \phi_{1}=\rho +d_{A},\\ & \phi_{2}=-\beta_{H}c,\\ & \phi_{3}={\left(\rho -d_{A}\right)}^{2},\\ & \phi_{4}=2\beta_{H}c\left(d_{A}+\rho \left(2\eta -1\right)\right),\\ & \phi_{5}={\left(\beta_{H}c\right)}^{2}. \end{array}$$

Let $\sqrt{\phi_{3}{ℛ}_{0_{A}}^{2}+\phi_{4}{ℛ}_{0_{A}}+\phi_{5}}=\phi_{6}{ℛ}_{0_{A}}+\phi_{7}$, then, (40) becomes

(42) $$\begin{array}{c} \mu_{H}=\frac{\left(\phi_{6}-\phi_{1}\right){ℛ}_{0_{A}}+\left(\phi_{7}-\phi_{2}\right)}{2{ℛ}_{0_{A}}}. \end{array}$$

Substituting (42) into the expression for *ℛ*_0_*B*__, we have

(43) $$\begin{array}{c} {ℛ}_{0_{B}}^{2}=\frac{4{ℛ}_{0_{S}}{ℛ}_{0_{A}}^{2}\beta_{P}N_{E}\gamma \Lambda_{H}}{\mu_{M}P_{0}\left[{\left(\left(\phi_{6}-\phi_{1}\right){ℛ}_{0_{A}}+\left(\phi_{7}-\phi_{2}\right)\right)}^{2}+2d_{A}{ℛ}_{0_{A}}\left(\left(\phi_{6}-\phi_{1}\right){ℛ}_{0_{A}}+\left(\phi_{7}-\phi_{2}\right)\right)\right]}. \end{array}$$

Differentiating *ℛ*_0_*B*__ partially with respect to *ℛ*_0_*A*__ yields

(44) $$\begin{array}{c} \frac{\partial {ℛ}_{0_{B}}}{\partial {ℛ}_{0_{A}}}=\frac{4{ℛ}_{0_{S}}{ℛ}_{0_{A}}\beta_{P}N_{E}\gamma \Lambda_{H}\left({ℛ}_{0_{A}}\left(\phi_{7}-\phi_{2}\right)\left(\phi_{6}-\phi_{1}-d_{A}\right)+{\left(\phi_{7}-\phi_{2}\right)}^{2}\right)}{\mu_{M}P_{0}{ℛ}_{0_{B}}{\left[{\left(\left(\phi_{6}-\phi_{1}\right){ℛ}_{0_{A}}+\left(\phi_{7}-\phi_{2}\right)\right)}^{2}+2d_{A}{ℛ}_{0_{A}}\left(\left(\phi_{6}-\phi_{1}\right){ℛ}_{0_{A}}+\left(\phi_{7}-\phi_{2}\right)\right)\right]}^{2}}. \end{array}$$

Now, whenever (44) is greater than zero, an increase in HIV/AIDS cases results in an increase of schistosomiasis cases in the community. If (44) is equal to zero, this implies that HIV/AIDS cases have no effect on the transmission dynamics of schistosomiasis. Setting *ℛ*_0_*B*__ = 1 and expressing *μ*_*H*_ as the subject of formula, we have

(45) $$\begin{array}{c} \mu_{H}=\frac{-d_{B}\theta_{1}{ℛ}_{0_{B}}+\sqrt{{\left(\theta_{1}d_{B}{ℛ}_{0_{B}}\right)}^{2}+4\theta_{2}}}{2\theta_{1}{ℛ}_{0_{B}}}, \end{array}$$

where *θ*_1_ = *μ*_*M*_ *P*_0_ and *θ*_2_ = *μ*_*M*_ *P*_0_ *β*_*P*_ *N*_*E*_ *γ*Λ_*H*_ *ℛ*_0_*S*__. Consider $\sqrt{{\left(\theta_{1}d_{B}{ℛ}_{0_{B}}\right)}^{2}+4\theta_{2}}=\theta_{3}{ℛ}_{0_{B}}+\theta_{4}$ such that (*θ*_3_ − *d*_*B*_ *θ*_1_)*ℛ*_0_*B*__ + *θ*_4_ > 0. Then, *ℛ*_0_*A*__ expressed in terms of *ℛ*_0_*B*__ reads

(46) $$\begin{array}{c} {ℛ}_{0_{A}}=\frac{2\theta_{1}\beta_{H}c\left(\kappa {ℛ}_{0_{B}}^{2}+\theta_{4}{ℛ}_{0_{B}}\right)}{h_{1}{ℛ}_{0_{B}}^{2}+h_{2}{ℛ}_{0_{B}}+h_{3}}, \end{array}$$

where

(47) $$\begin{array}{cc} & h_{1}={\left(\theta_{3}-d_{B}\theta_{1}\right)}^{2}+4\rho d_{A}\theta_{1}^{2}+2\theta_{1}\left(\theta_{3}-d_{B}\theta_{1}\right)\left(\rho +d_{A}\right)>0,\\ & h_{2}=2\theta_{4}\left(\theta_{3}-d_{B}\theta_{1}\right)+2\theta_{1}\theta_{4}\left(\rho +d_{A}\right)>0,\\ & h_{3}=\theta_{4}^{2}>0,\\ & \kappa_{1}=\theta_{3}+\theta_{1}\left(2\eta \rho +2d_{A}-d_{B}\right)>0. \end{array}$$

Partially differentiating *ℛ*_0_*A*__ with respect to *ℛ*_0_*B*__ yields

(48) $$\begin{array}{c} \frac{\partial {ℛ}_{0_{A}}}{\partial {ℛ}_{0_{B}}}=\frac{\left(\kappa_{1}h_{2}-\theta_{4}h_{1}\right){ℛ}_{0_{B}}^{2}+2\kappa_{1}h_{3}{ℛ}_{0_{B}}+\theta_{4}h_{3}}{{\left(h_{1}{ℛ}_{0_{B}}^{2}+h_{2}{ℛ}_{0_{B}}+h_{3}\right)}^{2}}. \end{array}$$

**Figure 2:**
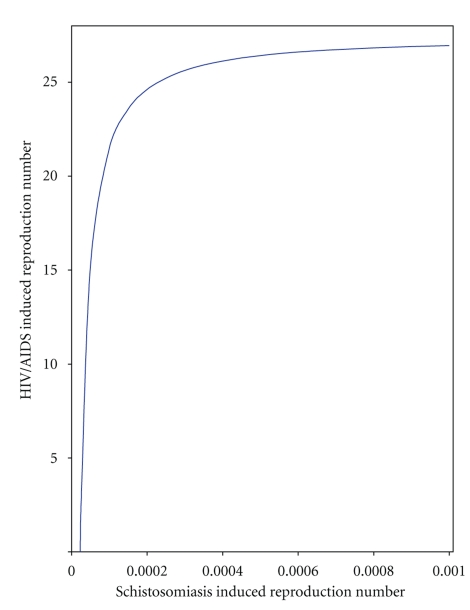
Relationship between the HIV/AIDS and the schistosomiasis basic reproduction numbers.

Thus, whenever *κ*_1_ *h*_2_ ≥ *θ*_4_ *h*_1_, (48) is strictly positive meaning that schistosomiasis enhances HIV infection as a damaged urethra has increased chances of HIV entering the blood stream. The relationship between the HIV/AIDS basic reproduction number and the schistosomiasis basic reproduction number is illustrated graphically in Figure 2 using parameter values from Table 1.

The graph in Figure 2 shows that an increase in the schistosomiasis-induced basic reproduction number results in an increase of the HIV/AIDS-induced basic reproduction number, suggesting that infection by schistosomiasis enhances the chances of HIV infection per sexual contact. This is as a result of the eggs of the parasites causing injury in the reproductive organs which enhance the transmission of sexually transmitted diseases such as HIV/AIDS and Gonorrhoea [51]. Thus, schistosomiasis control has a positive impact in controlling the transmission dynamics of HIV/AIDS.

**Impact of Schistosomiasis Treatment on HIV/AIDS**

Expressing *ℛ*_0*B*_ in terms of *ℛ*_*B*_, we obtain

(49) $$\begin{array}{c} {ℛ}_{0_{B}}=\frac{\left(\mu_{H}+\omega +d_{B}\right){ℛ}_{B}}{\left(\mu_{H}+d_{B}\right)}. \end{array}$$

Substituting (49) into (46) yields

(50) $$\begin{array}{c} \begin{array}{c} {ℛ}_{0_{A}}=\frac{2\beta_{H}c\theta_{1}{ℛ}_{B}\left[\left(\mu_{H}+\omega +d_{B}\right)(\theta_{4}\left(\mu_{H}+d_{B}\right)+\left(\mu_{H}+\omega +d_{B}\right)\kappa_{1}{ℛ}_{B}\right]}{{\left(\mu_{H}+\omega +d_{B}\right)}^{2}h_{1}{ℛ}_{B}^{2}+\left(\mu_{H}+d_{B}\right)\left(\mu_{H}+\omega +d_{B}\right)h_{2}{ℛ}_{B}+{\left(\mu_{H}+d_{B}\right)}^{2}h_{3}}. \end{array} \end{array}$$

Partially differentiating *ℛ*_0_*A*__ with respect to *ω*, we have

(51) $$\frac{\partial {ℛ}_{0_{A}}}{\partial \omega }=-\frac{2\beta_{H}c\theta_{1}k_{3}}{k_{4}}\left[\Theta -1\right],$$

where Θ = *k*_1_ *k*_2_/*k*_3_ *k*_4_, with

(52) $$\begin{array}{cc} & k_{1}={ℛ}_{B}\left[\left(\mu_{H}+d_{B}\right)h_{2}+2\zeta h_{1}{ℛ}_{B}\right],\\ & k_{2}=\zeta {ℛ}_{B}\left[\left(\mu_{H}+d_{B}\right)\theta_{4}+\zeta \kappa_{1}{ℛ}_{B}\right],\\ & k_{3}={ℛ}_{B}\left[\left(\mu_{H}+d_{B}\right)\theta_{4}+2\zeta \kappa_{1}{ℛ}_{B}\right],\\ & k_{4}=\zeta {ℛ}_{B}\left[\zeta h_{1}{ℛ}_{B}+\left(\mu_{H}+d_{B}\right)h_{2}\right]+h_{3},\\ & \zeta =\left(\mu_{H}+\omega +d_{B}\right). \end{array}$$

Since *ℛ*_0_*A*__ is a decreasing function of *ω*, schistosomiasis treatment will have a positive impact on the dynamics of HIV/AIDS if Θ > 1, no impact if Θ = 1, and a negative impact if Θ < 1. We summarize the result in lemma 5.

**Lemma 5**

Schistosomiasis (bilharzia) treatment for model system (5) only, will have

- a positive impact on schistosomiasis and HIV/AIDS coinfection control if Θ > 1,
- no impact on schistosomiasis and HIV/AIDS coinfection control if Θ = 1,
- a negative impact on schistosomiasis and HIV/AIDS coinfection control if Θ < 1.

The synergy between HIV and other diseases such as schistosomiasis provides more opportunities to combat HIV/AIDS by treating its coinfections with these other diseases.

### 4.2. Global Stability of the Disease-Free Equilibrium (*𝒰*_0_)

We shall use the following theorem of Castillo-Chavez et al. [50] in the sequel.

**Theorem 8 (see [50])**

If system (5) can be written in the form

(53) $$\begin{array}{c} \frac{dX}{dt}=F\left(x,Z\right),\\ \frac{dZ}{dt}=G\left(X,Z\right),\;G\left(x,0\right)=0, \end{array}$$

where *X* ∈ ℝ^*m*^ denotes (its components) the number of uninfected individuals, *Z* ∈ ℝ^*n*^ denotes (its components) the number of infected individuals including latent, infectious, and so forth, **U**_0_ = (x*, 0) denotes the disease-free equilibrium of the system. Assume that (i) for *dX*/*dt* = *F*(*X*, 0),X* is globally asymptotically stable, (ii) $G(X,Z)=AZ-\hat{G}(X,Z)$, $\hat{G}(X,Z)\geq 0$ for (*X*, *Z*) ∈ *𝒟*, where *A* = *D*_*Z*_ *G*(X*, 0) is an *M*-matrix (the off-diagonal elements of *A* are nonnegative) and *𝒟* is the region where the model makes biological sense. Then the fixed point **U**_0_ = (x*, 0) is a globally asymptotic stable equilibrium of model system (5) provided *ℛ*_*H*_*B*__ < 1.

Applying Theorem 8 to model system (5) yields

(54) $$\begin{array}{cc} & \hat{G}\left(X,Y\right)\\ & =\left[\begin{array}{c} \hat{G_{1}}\left(X,Y\right)\\ \hat{G_{2}}\left(X,Y\right)\\ \hat{G_{3}}\left(X,Y\right)\\ \hat{G_{4}}\left(X,Y\right)\\ \hat{G_{5}}\left(X,Y\right)\\ \hat{G_{6}}\left(X,Y\right)\\ \hat{G_{7}}\left(X,Y\right)\\ \hat{G_{8}}\left(X,Y\right)\\ \hat{G_{9}}\left(X,Y\right)\\ \hat{G_{10}}\left(X,Y\right) \end{array}\right]=\left[\begin{array}{c} \delta \lambda_{H}I_{B}+\beta_{P}\left(\frac{P\left(t\right)\Lambda_{H}}{P_{0}\mu_{H}}-\frac{P\left(t\right)S_{H}\left(t\right)}{P_{0}+\epsilon P\left(t\right)}\right)\\ \lambda_{P}I_{H}+N_{H}\left(1-\frac{S_{H}}{N_{H}}\right)\\ \lambda_{P}A_{H}\\ \lambda_{P}A_{H_{T_{A}}}\\ -\lambda_{P}I_{H}-\delta \lambda_{H}I_{B}\\ -\lambda_{P}A_{H}\\ -\lambda_{P}A_{H_{T_{A}}}\\ 0\\ \beta_{M}\left(\frac{M\Lambda_{S}}{M_{0}\mu_{S}}-\frac{MS_{S}}{M_{0}+\epsilon M}\right)\\ 0 \end{array}\right]. \end{array}$$

The fact that $\hat{G_{4}}(X,Y)<0$, $\hat{G_{5}}(X,Y)<0$, and $\hat{G_{6}}(X,Y)<0$ implies that $\hat{G}(X,Y)$ may not be greater or equal to zero. Consequently, *𝒰*_0_ may not be globally asymptotically stable for *ℛ*_*H*_*B*__ < 1. This suggests the possible existence of multiple equilibria.

### 4.3. Endemic Equilibria and Its Stability

For model system (5), there are three possible endemic equilibria: the case where there is HIV only, the case where there is schistosomiasis only (which have been discussed in Section 3), and the case when both schistosomiasis and HIV coexist.

#### 4.3.1. Interior Endemic Equilibrium

This occurs when both infections coexist in the community. The interior equilibrium is given by

(55) $$\begin{array}{cc} {𝒰}_{3}^{*} & =(S_{H}^{***},I_{B}^{***},I_{H}^{***},A_{H}^{***},A_{HT_{A}}^{***},I_{H_{B}}^{***},A_{H_{B}}^{***},\\ & \;\;A_{HT_{B}}^{***}M^{***},S_{S}^{***},I_{S}^{***},P^{***}). \end{array}$$

The local asymptotic stability of this endemic equilibrium can be analyzed using the centre manifold theory similar to the analysis of *𝒰*_1_* and *𝒰*_2_*, but it is not done here to avoid repetition. Thus, we claim the following result for the stability of *𝒰*_1_* and *𝒰*_2_*.

**Theorem 9**

If *ℛ*_*H*_*B*__ > 1 with *ℛ*_*B*_ > 1 and *ℛ*_*A*_ > 1, then, the endemic equilibrium point *𝒰*_3_ is locally asymptotically stable whenever *ℛ*_*H*_*B*__ > 1.

## 5. Numerical Simulations

In order to illustrate the results of the foregoing analysis, numerical simulations of the full HIV-schistosomiasis model are carried out, using parameter values given in Table 1. The scarcity of data on HIV schistosomiasis codynamics limits our ability to calibrate, but, for the purpose of illustration, other parameter values are assumed. These parsimonious assumptions reflect the lack of information currently available on the coinfection of the two diseases. 

**Figure 3:**
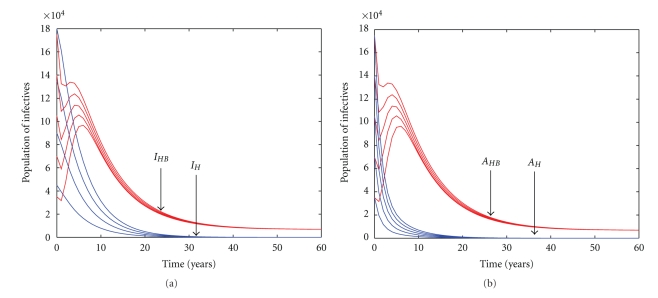
Numerical results of model system (5) showing time series plots of infectives either singly infected with HIV or dually infected with HIV and schistosomiasis for both cases (i.e., either displaying clinical symptoms of AIDS or not), using various initial conditions and parameter values from Table 1.

Figure 3 depicts the effects of schistosomiasis on the dynamics of HIV in the community. The time series plots in Figure 3 suggest that the presence of schistosomiasis in the community might increase the prevalence of HIV/AIDS. These numerical results are in agreement with our analytical results. We note that *I*_*H*_ and *A*_*H*_ are not reflecting the disease-free equilibrium, and the convergence is simply due to scale.

## 6. Summary and Conclusion

While schistosomiasis is the second most prevalent neglected tropical disease after hookworm infection (192 million cases worldwide) [5], HIV on the other hand which has killed more than 25 million people since first recognized in 1981 currently affects 33.4 million people, with deaths due to HIV/AIDS-related illnesses standing at about 2 million in 2008 [6]. A mathematical model for investigating the coinfection of schistosomiasis and HIV/AIDS is derived. Comprehensive and qualitative mathematical techniques were used to analyze steady states of the model. The disease-free equilibrium is shown to be locally asymptotically stable when the associated epidemic threshold known as the basic reproduction number for the model is less than unity. Center manifold theory is used to show that the schistosomiasis-only and HIV/AIDS-only endemic equilibria are locally asymptotically stable when the associated reproduction numbers are greater than unity. The impact of schistosomiasis and its treatment on the dynamics of HIV/AIDS is also investigated. Numerical results are provided to illustrate some of analytical results.

In this study, the impact of schistosomiasis and its treatment on the transmission dynamics of HIV/AIDS in the community is investigated by formulating a mathematical model that incorporates both key epidemiological parameters of both schistosomiasis and HIV/AIDS. Mathematical and numerical analysis of the model suggests that schistosomiasis may increase the prevalence of HIV/AIDS in the community. Analysis of the impact of schistosomiasis treatment has shown that the impact of this form of treatment depends on the sign of a certain threshold parameter Θ, and for Θ > 1, schistosomiasis treatment will have a positive impact, for Θ = 1, no impact, and for Θ < 1, a negative impact on controlling the co-interaction of the two diseases. We, however, note that from schistosomiasis and HIV/AIDS epidemiology, realistic parameter values always yield 1 < Θ. Consequently, schistosomiasis treatment will always have a positive impact on the control of both schistosomiasis and HIV/AIDS codynamics. Thus, schistosomiasis treatment can reduce the burden of schistosomiasis and HIV/AIDS coinfection in areas of extreme poverty, especially among the rural poor and some disadvantaged urban populations since it is less expensive and usually available in government clinics and hospitals. This outcome highlights the fact that global public health challenges require comprehensive and multipronged approaches to dealing with them. Current efforts that focus on a single infection at a time may be losing substantial rewards of dealing synergistically and concurrently with multiple infectious diseases [7].

## Figures and Tables

**Table 1:** Model parameters and their interpretations.

Parameter	Symbol	Value	Source
Recruitment rate for humans	Λ_*H*_	100,000 yr^−1^	[40]
Natural mortality rate for humans	*μ*_*H*_	0.02 yr^−1^	[39, 40]
Natural rate of progression to AIDS	*ρ*	0.125 yr^−1^	[40]
AIDS-related death rate	*d*_*A*_	0.333 yr^−1^	[39]
Schistosomiasis-related death rate	*d*_*B*_	0.00201 yr^−1^	Assume
Product of effective contact rate			
for HIV infection and probability			
of HIV transmission per contact	*β*_*H*_ *c*	0.011–0.95 yr^−1^	[39, 40]
Enhancement factor of schistosomiasis			
to HIV infection	*δ*	1.001 yr^−1^	[39]
Modification parameter	*σ*	1.001 yr^−1^	[39]
Treatment rate	*α*	0.33 yr^−1^	Assume
Recruitment rate for snails	Λ_*S*_	10 yr^−1^	[39]
Natural mortality rate from snails	*μ*_*S*_	0.072 yr^−1^	Assume
Saturation constant for cercariae	*P*_0_	10^7^	[39]
Saturation constant for miracidia	*M*_0_	10^8^	[39]
Limitation of the growth velocity	*ϵ*	100	[39]
Number of eggs excreted by humans	*N*_*E*_	500	[39]
Mortality rate for cercariae	*μ*_*P*_	0.504 yr^−1^	[39]
Mortality rate for miracidia	*μ*_*M*_	0.65 yr^−1^	Assume
Snail disease induced death rate	*d*_*S*_	0.08 yr^−1^	Assume
Rate at which eggs successfully			
become miracidia	*γ*	0.835 yr^−1^	[39]
Rate at which sporocysts successfully			
become cercariae	*θ*	0.9 yr^−1^	[39]
Modification parameter	*κ*	0.4	[41]
Modification parameter	*η*	1.25	Assume
Modification parameter	*τ*	0.001	Assume
Rate of recovery from schistosomiasis	*ω*, *ω*_1_	0.56	Assume

## Appendix

In order to establish the conditions for the existence of a bifurcation, we use Theorem 10 proven in [47].

Theorem 10 Consider the following general system of ordinary differential equations with a parameter *ϕ*:

(A.1) $$\begin{array}{c} \begin{array}{c} \frac{dx}{dt}=f\left(x,\phi \right),\;f:{\mathbb{R}}^{n}\times \mathbb{R}\to \mathbb{R},\;f\in {\mathbb{C}}^{2}\left({\mathbb{R}}^{n}\times \mathbb{R}\right), \end{array} \end{array}$$

where 0 is an equilibrium of the system that is *f*(0, *ϕ*) = 0 for all *ϕ*, and assume that

(A1) *A* = *D*_*x*_ *f*(0,0) = ((∂*f*_*i*_/∂*x*_*j*_)(0,0)) is linearization of system (A.1) around the equilibrium 0 with *ϕ* evaluated at 0. Zero is a simple eigenvalue of *A*, and other eigenvalues of *A* have negative real parts,
(A2) matrix *A* has a right eigenvector *u* and a left eigenvector *v* corresponding to the zero eigenvalue.

Let *f*_*k*_ be the *K*th component of *f* and

(A.2) $$\begin{array}{c} a=\overset{n}{\underset{k,i,j=1}{\sum }}v_{k}u_{i}u_{j}\frac{\partial^{2}f_{k}}{\partial x_{i}\partial x_{j}}\left(0,0\right),\\ b=\overset{n}{\underset{k,i=1}{\sum }}v_{k}u_{i}\frac{\partial^{2}f_{k}}{\partial x_{i}\partial \phi }\left(0,0\right). \end{array}$$

The local dynamics of (A.1) around 0 are totally governed by *a* and *b*.

- *a* > 0, *b* > 0. When *ϕ* < 0 with |*ϕ*| ≪ 1, 0 is locally asymptotically stable, and there exists a positive unstable equilibrium; when 0 < *ϕ* ≪ 1, 0 is unstable and there exists a negative and locally asymptotically stable equilibrium.
- *a* < 0, *b* < 0. When *ϕ* < 0 with |*ϕ*| ≪ 1, 0 is unstable and when 0 < *ϕ* ≪ 1, asymptotically stable, and there exists a positive unstable equilibrium.
- *a* > 0, *b* < 0. When *ϕ* < 0 with |*ϕ*| ≪ 1, 0 is unstable, and there exists a locally asymptotically stable negative equilibrium; when 0 < *ϕ* ≪ 1, 0 is stable, and a positive unstable equilibrium appears.
- *a* < 0, *b* > 0. When *ϕ* changes from negative to positive, 0 changes its stability from stable to unstable. Correspondingly, a negative equilibrium becomes positive and locally asymptotically stable.

Computations of *a* and *b*
For system (16), the associated nonzero partial derivatives of *F* associated with *a* at the disease-free equilibrium is given by

(A.3) $$\begin{array}{cc} \frac{\partial^{2}f_{2}}{\partial x_{2}\partial x_{3}} & =\frac{\partial^{2}f_{2}}{\partial x_{3}\partial x_{2}}=-\frac{\beta_{H}^{*}c\left(1+\eta \right)\mu_{H}}{\Lambda_{H}},\\ \frac{\partial^{2}f_{2}}{\partial x_{2}^{2}} & =-\frac{2\beta_{H}^{*}c\mu_{H}}{\Lambda_{H}},\\ \frac{\partial^{2}f_{2}}{\partial x_{2}\partial x_{4}} & =\frac{\partial^{2}f_{2}}{\partial x_{4}\partial x_{2}}=-\frac{\beta_{H}^{*}c\left(1+\kappa \right)\mu_{H}}{\Lambda_{H}},\\ \frac{\partial^{2}f_{2}}{\partial x_{3}^{2}} & =-\frac{2\beta_{H}^{*}c\eta \mu_{H}}{\Lambda_{H}},\\ \frac{\partial^{2}f_{2}}{\partial x_{3}\partial x_{4}} & =\frac{\partial^{2}f_{2}}{\partial x_{4}\partial x_{3}}=-\frac{\beta_{H}^{*}c\left(\eta +\kappa \right)\mu_{H}}{\Lambda_{H}},\\ \frac{\partial^{2}f_{2}}{\partial x_{4}^{2}} & =-\frac{2\beta_{H}^{*}c\kappa \mu_{H}}{\Lambda_{H}}. \end{array}$$

From (A.3), it follows that

(A.4) $$a=-\frac{2\beta_{H}^{*}c\mu_{H}v_{2}}{\Lambda_{H}}\left(u_{2}+u_{3}+u_{4}\right)\left(u_{2}+\eta u_{3}+\kappa u_{4}\right)<0.\;$$

For the sign of *b*, it is associated with the following nonvanishing partial derivatives of *F*:

(A.5) $$\begin{array}{c} \frac{\partial^{2}f_{2}}{\partial x_{2}\partial \beta_{H}^{*}}=c,\;\frac{\partial^{2}f_{2}}{\partial x_{3}\partial \beta_{H}^{*}}=c\eta ,\;\frac{\partial^{2}f_{2}}{\partial x_{4}\partial \beta_{H}^{*}}=c\kappa , \end{array}$$

from which it follows that

(A.6) $$b=c\left(u_{2}+\eta u_{3}+\kappa u_{4}\right)v_{2}>0.$$

Thus, *a* < 0 and *b* > 0 and from Theorem 10 item (iv), the result follows.
